# Antioxidant, Biomolecule Oxidation Protective Activities of *Nardostachys jatamansi* DC and Its Phytochemical Analysis by RP-HPLC and GC-MS

**DOI:** 10.3390/antiox4010185

**Published:** 2015-03-12

**Authors:** Sakina Razack, Kandikattu Hemanth Kumar, Ilaiyaraja Nallamuthu, Mahadeva Naika, Farhath Khanum

**Affiliations:** 1 Biochemistry and Nanosciences Division, Defence Food Research Laboratory, Mysore-570011, India; E-Mails: razacksakina@yahoo.co.in (S.R.); kandikattu.hemanth@gmail.com (K.H.K.); nilaiyaraja@gmail.com (I.N.); 2 Applied Nutrition Division, Defence Food Research Laboratory, Mysore-570011, India; E-Mail: mnaika@rediffmail.com

**Keywords:** antioxidant activity, DNA damage protective, GC-MS, *Nardostachys jatamansi*, phytochemical analysis, RP-HPLC

## Abstract

The study aimed at analyzing the metabolite profile of *Nardostachys jatamansi* using RP-HPLC, GC-MS and also its antioxidant, biomolecule protective and cytoprotective properties. The 70% ethanolic extract of *Nardostachys jatamansi* (NJE) showed the presence of polyphenols and flavonoids (gallic acid, catechin, chlorogenic acid, homovanillin, epicatechin, rutin hydrate and quercetin-3-rhamnoside) analyzed by RP-HPLC, whereas hexane extract revealed an array of metabolites (fatty acids, sesquiterpenes, alkane hydrocarbons and esters) by GC-MS analysis. The antioxidant assays showed the enhanced potency of NJE with a half maximal inhibitory concentration (IC_50_) value of 222.22 ± 7.4 μg/mL for 2,2-diphenyl-1-picrylhydrazyl (DPPH), 13.90 ± 0.5 μg/mL for 2,2′-azino-bis(3-ethyl benzothiazoline-6-sulfonic acid) diammonium salt (ABTS), 113.81 ± 4.2 μg/mL for superoxide, 948 ± 21.1 μg/mL for metal chelating and 12.3 ± 0.43 mg FeSO_4_ equivalent/g of extract for ferric reducing antioxidant power assays and was more potent than hexane extract. NJE effectively inhibited 2,2′-azobis(2-methylpropionamidine) dihydrochloride (AAPH)-induced oxidation of biomolecules analyzed by pBR322 plasmid DNA damage, protein oxidation of bovine serum albumin and lipid peroxidation assays. The observed effects might be due to the high content of polyphenols, 53.06 ± 2.2 mg gallic acid equivalents/g, and flavonoids, 25.303 ± 0.9 mg catechin equivalents/g, of NJE compared to the hexane fraction. Additionally, the extract abrogated the protein, carbonyl, and ROS formation, and NJE showed cytotoxicity in SH-SY5Y neuronal cells above 75 μg/mL. Thus, the study suggests that the herb unequivocally is a potential source of antioxidants and could aid in alleviating oxidative stress-mediated disorders.

## 1. Introduction

Reactive oxygen species (ROS) comprise free radicals, such as superoxide anion (O_2_^−^) and hydroxyl (HO**^·^**) radicals, and non-free radical species, such as H_2_O_2_ and singlet oxygen (^1^O_2_), and these comprise different forms of activated oxygen [1,2,3]. ROS are known to damage cellular membranes by inducing lipid peroxidation, damage DNA, proteins and lipids [4]. Thus, excessive production of free radicals/ROS leads to oxidation of biomolecules, and this has been implicated in the etiology of several human diseases, such as atherosclerosis, ischemia reperfusion injury, ageing and liver-related diseases [5,6]. To protect the cells and organ systems of the body from free radicals/ROS, the human body has evolved a highly sophisticated and complex antioxidant protection system. Antioxidants are capable of stabilizing, or deactivating, free radicals before they damage cells. The body’s antioxidant defense system comprises endogenous antioxidant enzymes, *viz*. superoxide dismutase, glutathione peroxidase, glutathione reductase, catalase, *etc.*, that catalyze free radical quenching reactions. In addition, dietary antioxidants, like ascorbic acid (vitamin C), tocopherols and tocotrienols (vitamin E), carotenoids and other low molecular weight compounds, such as glutathione and lipoic acid, also aid in stabilizing free radicals [7].

Plant-derived substances, collectively called phytonutrients or phytochemicals, are known for their antioxidant activity [7]. Consumption of natural antioxidants is associated with reduced risks of cancer, cardiovascular disease, diabetes and other diseases associated with ageing [8,9]. In recent years, consumption of the natural phytochemicals present in berry crops, teas, herbs, oilseeds, beans, fruits and vegetables have markedly increased [10,11]. The oxidative DNA damage plays a significant role in mutagenesis, cancer, aging and other human pathologies [12,13]. The best strategy to counter oxidative stress is supplementation of food rich in herbal bioactives [14].

*Nardostachys jatamansi* DC is a member of the plant family, *Valerianaceae*, is a perennial herb growing to about 10–60 m in height and is commonly referred to as Indian spikenard. It is found in the alpine Himalayas from Punjab to Sikkim and Bhutan at an altitude of 3000–5000 m. The plant has been reported to possess antianxiety, hepatoprotective, cardioprotective, anticonvulsant, antiparkinsons, antidepressant and antimicrobial activities [15,16,17,18,19,20,21,22,23].

*Nardostachys jatamansi* has been attributed with exorbitant medicinal properties, and it was our interest to analyze the 70% ethanolic and hexane extracts for their *in vitro* antioxidant activities, with the former extract comprising both polar and non-polar metabolites and the latter exclusively non-polar. Scarce literature is available with regards to the antioxidant and biomolecules protective properties of the 70% ethanolic extract and also with regard to the hexane extract. Hence, the rationale of the present study was to characterize the 70% ethanol and hexane extracts of *N. jatamansi* by RP-HPLC and GC-MS analysis and to assess the extract for its antioxidant potency, quantification of polyphenols and flavonoids and, further, to verify the ability of the extract to inhibit the oxidation of biomolecules to understand the possible role of the antioxidant activity of the herb.

## 2. Experimental Section

### 2.1. Chemicals and Reagents

2,2′-azino-bis(3-ethyl benzothiazoline-6-sulfonic acid) diammonium salt (ABTS), 2,2′-azobis(2-methylpropionamidine) dihydrochloride (AAPH), agarose, ethidium bromide, bovine serum albumin, gallic acid, catechin, homovanillic acid, epicatechin, chlorogenic acid, rutin hydrate and quercetin-3-rhamnoside were purchased from Sigma-Aldrich, St. Louis, MO, USA. pBR322 plasmid DNA was purchased from Genetix, Bangalore. India. The other chemicals and reagents were of analytical grade and were procured from Merck, Bangalore, India.

### 2.2. Plant Material and Preparation of the Extract

The root material was procured from a local supplier at Mysore, India. The plant was identified by Dr. K. Madhava Chetty, Botanist, Department of Botany, Sri Venkateswara University, Tirupati, India. The voucher specimen (Herbarium Accession Number 1911) was deposited in the herbarium, Department of Botany, Sri Venkateswara University, Tirupati, India. The roots were washed thoroughly with distilled water to remove the adhering sand particles and shade dried. The thoroughly dried roots were powdered and extracted by shaking with 70% ethanol and hexane with a plant material:solvent ratio of 1:10, filtered through Whatmann filter paper No. 1 (Sigma-Aldrich, St. Louis, MO, USA), and were evaporated to dryness under vacuum on a rotary evaporator (Heidolph rotacool, Germany) into a thick residue. The 70% ethanol fraction was subsequently lyophilized (Lyolab, Hyderabad, India), and the powder was used for analysis. The yield of the 70% ethanol fraction (NJE) was 7.4% and the hexane fraction (NJH) was 4.2%.

### 2.3. Metabolite Profile of N. jatamansi

#### 2.3.1. Reversed Phase-HPLC Analysis of the 70% Ethanol Fraction

Phenolic compounds of NJE were identified using a diode array detector (JASCO Pu-1580 HPLC system, JASCO Inc., Easton, MD, USA) on a reverse phase C_18_ column (150 × 4.5 mm, JASCO Inc.). The mobile phase was with two solvents: 0.1% formic acid in water (A) and 100% methanol (B). The total run time was 60 min. The eluting compounds were detected by monitoring at 270 nm. The phenolic compounds were identified by comparing the retention time (RT) of the unknowns with the standards [24]. The polyphenols were partially extracted from the sample using the solid-phase extraction method on a C_18_ reverse phase sep-pak column (Catologue Number: WAT094226, HLB3CC, Waters India Private Limited, Bengaluru, Karnataka, India), and the eluant obtained was injected into reversed phase-HPLC for analysis. The lyophilized plant extract (1 mg/mL) was redissolved in 0.1% formic acid in water, and 20 μL of the sample and the standards were injected for analysis. The standards injected include gallic acid (RT-9.98), catechin (RT-18.17), chlorogenic acid (RT-22.02), homovanillin (RT-23.12), epicatechin (RT-24.09), rutin hydrate (RT-37.33) and quercetin-3-rhamnoside (RT-40.61).

#### 2.3.2. Gas Chromatography and Mass Spectrometry Analysis (GC-MS-HT-TOF) of the Hexane Fraction

The metabolite profiling of NJH was carried out by GC–MS analysis on an Agilent 7890 gas chromatograph (Agilent Technologies, Santa Clara, CA, US) coupled with LECO Corporation (St. Joseph, MI, USA) with the following conditions: the column used was 29.3 m × 0.7 m, 320 μm, capillary column −29.300 m, operating in an electron ionization mode at 70 eV; with helium as a carrier gas at a constant flow of 1.50 mL/min and an injection volume of 1.0 μL; injector temperature 250 °C; ion source temperature 200 °C. The oven temperature was initially 70 °C for 1 min, increased to 160 °C at a rate of 4 °C/min and increased finally to 320 °C for 15 min at a rate of 3 °C/min. The mass spectrometer was operated at an acquisition rate of 10 spectra/s, and the scan range was set between 50 and 600 m/z. The detector voltage was set to 1450 V and the electron energy to −70 V.

### 2.4. Phytochemical Screening of the Plant Extract

#### 2.4.1. Determination of Total Phenolic Content

The total polyphenols were estimated by the Folin–Ciocalteu method [25]. Briefly, to different concentrations of the plant extract (100–1000 μg/mL), 2.5 mL of Folin-Ciocalteu reagent (1:10) and 2 mL of 7% Na_2_CO_3_ were added and incubated at room temperature for 90 min, and the absorbance was measured spectrophotometrically at 765 nm (Shimadzu, Kyoto, Japan). Gallic acid (10–100 μg/mL) was used as a standard, and the amount of total polyphenolic content was expressed as mg gallic acid equivalent per gram extract (mg GAE/g).

#### 2.4.2. Determination of Total Flavonoid Content

Briefly, to different concentrations of plant extracts (100–1000 μg/mL), 75 μL of 5% NaNO_2_ and 150 μL of 10% AlCl_3_ were added and incubated at room temperature for 5 min. Then, 500 μL of 1 M NaOH followed by 775 μL of water were added, vortexed and the absorbance measured at 510 nm [26]. Catechin (10–100 μg/mL) was used as the standard, and the amount of total flavonoids was expressed as mg of catechin equivalents per gram extract (mg CE/g).

### 2.5. In Vitro Free Radical Scavenging and Antioxidant Activities

#### 2.5.1. DPPH Radical Scavenging Activity

The free radical scavenging activity of NJE and NJH were measured by the 2,2-diphenyl-1-picrylhydrazyl (DPPH) assay [27]. Briefly, 500 μL of DPPH (0.5 mM in methanol) solution was mixed with different amounts of sample (100–1000 μg/mL), and the volume was made to 3 mL with methanol. The mixture was incubated in the dark for 45 min at room temperature, and the absorbance was recorded at 515 nm. Butylated hydroxyanisole (BHA; 10–100 μg/mL) was used as the standard antioxidant compound. Methanol was used as the blank. The percent inhibition was calculated as follows:

Percentage inhibition (%) = [*A*_C_ − *A*_S_/*A*_C_] × 100 (1)

where *A*_C_ is the absorbance of positive control and *A*_S_ is the absorbance of the sample.

#### 2.5.2. ABTS Radical Cation Decolorization Assay

The ABTS (2,2′-azino-bis(3-ethyl benzothiazoline-6-sulfonic acid) diammonium salt) radical cation scavenging activity of NJE/NJH was assayed [28]. The working solution was prepared by mixing two stock solutions of 14 mM ABTS solution and 4.9 mM potassium persulfate solution in equal amounts and allowed to react for 12–16 h at room temperature in the dark. This solution (5 mL) was diluted with absolute ethanol until the OD value reached 0.70 ± 0.02 at 734 nm. The working solution was thoroughly mixed at room temperature, and 1 mL of this solution was mixed with different concentrations of plant extracts (10–100 μg/mL). Additionally, the scavenging capacity of the extract for ABTS^+^ was calculated and expressed as the percent inhibition. The IC_50_ value was calculated and compared with ascorbic acid (5–50 μg/mL), used as a standard.

#### 2.5.3. Metal Chelating

Briefly, to different concentrations of the extracts (100–2000 μg/mL), 50 μL of FeCl_2_ (2 mM) were added. The reaction was initiated by the addition of 200 μL of ferrozine (2 mM); the mixture was vortexed and left at room temperature for 10 min. Absorbance was then measured spectrophotometrically at 562 nm [29]. The % of inhibition of the ferrozine-Fe^2+^ complex was calculated by:

% Inhibition = [(*A*_0_ − *A*_1_)/*A*_0_] × 100 (2)

where *A*_0_ is the absorbance of the control and *A*_1_ is the absorbance of the samples and standards. The ability of the extracts to chelate ferrous ion was compared with EDTA (100 μg/mL), used as a standard.

#### 2.5.4. Superoxide Radical Scavenging Activity

The superoxide radicals were generated in 16 mM Tris-HCl buffer (pH 8.0) containing 50 μM nitro blue tetrazolium (NBT), 78 μM NADH and different concentrations of plant extract (100–1000 μg/mL). The reaction was started by adding 10 μM PMS to the mixture. The reaction mixture was incubated at 25 °C for 5 min, and the absorbance was measured at 560 nm [30]. l-ascorbic acid (10–100 μg/mL) was used as the control. The percent inhibition of superoxide anion generation was calculated by using the following formula:

% Inhibition = [(*A*_0_ − *A*_1_)/*A*_0_] × 100 (3)

where *A*_0_ is the absorbance of the control and *A*_1_ is the absorbance of sample extract/standard.

#### 2.5.5. Ferric-Reducing Antioxidant Power Assay

The antioxidant capacity was determined by the ferric-reducing antioxidant power (FRAP) assay [31]. The stock solutions included 300 mM acetate buffer (3.1 g of CH_3_COONa and 16 mL of CH_3_OOH), pH 3.6, 10 mM TPTZ (2,4,6-tripyridyl-*s*-triazine) solution in 40 mM HCl and 20 mM FeCl_3_·6H_2_O solution. The fresh working solution was prepared by mixing 25 mL acetate buffer, 2.5 mL TPTZ and 2.5 mL FeCl_3_·6H_2_O. The temperature of the solution was raised to 37 °C before the assay. Plant extract (10–100 μg/mL) was allowed to react with 2850 μL of the FRAP solution for 30 min in the dark. Readings of the colored product (ferrous tripyridyltriazine complex) were measured at 593 nm.

### 2.6. Biomolecules Oxidation Assays

#### 2.6.1. AAPH-Induced Plasmid Nick Assay

Conversion of the supercoiled form of plasmid DNA to open circular and further linear form has been used as an index of DNA damage. The DNA damage protective activity of the NJE was assessed using pBR322 plasmid DNA [32]. Different concentrations of NJE (5, 10, 15 μg from a stock of 1 mg/mL) and plasmid DNA (0.5 μg) were incubated at room temperature followed by the addition of AAPH (200 mM). The reaction mixture was made up to 20 μL and incubated for one hour at 37 °C. The DNA samples were electrophoresed on 1% agarose gel, and the band intensities were analyzed using Easy win 32 software from Herolab (Herolab GmbH Laborgeraete, Wiesloch, Germany).

#### 2.6.2. Protein Oxidation

The oxidation of BSA (5 μg) in phosphate buffer was initiated by 200 mM AAPH, and the inhibitory properties of NJE were measured at a fixed concentration (1 mg/mL). After incubation for one hour at 37 °C, BHT (0.02%) was added to prevent further formation of peroxyl radicals, and the samples were assayed by SDS-PAGE [33].

#### 2.6.3. Thiobarbituric Acid-Reactive Substances Assay

Male Wistar albino rats weighing 200–220 g were housed under conventional conditions and were allowed free access to food and water. The experiments were carried out according to Committee for the Purpose of Control and Supervision of Experiments on Animals (CPCSEA) guidelines for the care and use of experiment animals approved by the Institutional Animal Ethics Committee. The rats were anaesthetized, and the liver and brain were removed and homogenized (10% w/v) in sodium phosphate buffer (pH, 7.4). Homogenates were then centrifuged at 5000× *g* for 15 min at 4 °C. Oxidative stress was induced using AAPH [34]. The reaction mixture was composed of liver/brain homogenate (15 mg protein), 120 mM AAPH and different concentrations of NJE (100–1000 μg/mL). The reaction mixture was incubated at 37 °C for 2 h, and the extent of lipid peroxidation of the liver homogenate in the presence and absence of the plant extract was evaluated by measuring the product of thiobarbituric acid reactive substances. After incubation, the reaction was terminated by adding 2% BHT followed by the addition of 1 mL of TCA (20% w/v) to the mixture. After centrifugation at 3000× *g* for 15 min, the supernatant was incubated with 1 mL of thiobarbituric acid (TBA, 0.67%) at 100 °C for 15 min, and the absorbance was measured at 532 nm. The data were expressed in terms of the percent inhibition.

% Inhibition = [*A*_C_ − *A*_S_/*A*_C_] × 100 (4)

where *A*_C_ is the absorbance of the control, and *A*_S_ is the absorbance of the sample.

#### 2.6.4. Estimation of Reactive Oxygen Species

The extent of inhibition of reactive oxygen species (ROS) was evaluated using 2′,7′-dichlorodihydro fluorescein diacetate (DCHF-DA). The brain and liver homogenates (10% w/v) were incubated with different concentrations of NJE (10–100 μg/mL) and gallic acid (1 mg/mL) as a standard. After incubation for half an hour, AAPH (120 mM), a peroxyl radical inducer, was added to all of the wells, except the control, and incubated for 30 min. Later, 10 μM of DCFH-DA was added and incubated for 60 min. The fluorescence of DCF (dichlorofluorescein) was measured at 492-nm excitation and 520-nm emission. Results were expressed as the percent of fluorescence [35].

#### 2.6.5. Estimation of Protein Carbonyls

The protein carbonyl content (PCC) of the rat liver homogenates in the presence and absence of NJE (10–100 μg/mL)/AAPH (120 mM) was evaluated [36]. One milliliter of 10 mM DNPH (dinitrophenyl hydrazine) in 2 N HCl was added to the reaction mixture (2 mg of protein), and samples were incubated for 1 h at room temperature. Then, 1 mL of trichloroacetic acid 10% was added to each reaction mixture and centrifuged at 3000× *g* for 10 min. The protein pellet was washed thrice with 2 mL of ethanol/ethyl acetate (1:1, v/v) and dissolved in 1 mL of guanidine hydrochloride (6 M, pH 2.3) and incubated for 10 min at 37 °C. The carbonyl content was calculated based on the molar extinction coefficient of DNPH (ε = 2.2 × 104 cm^−1^ M^−1^). The data were expressed in terms of the percent of inhibition.

### 2.7. Cytoprotective Effects

#### LDH Leakage Assay

The human neuroblastoma SH-SY5Y cells were procured from the National Center for Cell Sciences, Pune, India. Cells were seeded into 25-cm^2^ flasks in DMEM/F-12, 1:1 mixture supplemented with 10% fetal bovine serum, 2 mM l-glutamine, antibiotic and antimycotic solution and maintained in a humid atmosphere with 5% CO_2_ and 95% air at 37 °C. Media were changed every alternate day, and the experiment was carried out in serum-free media. Once the cells reached confluence, they were treated with NJE at different concentrations (10, 25, 50, 75, 100, 150, 200, 300, 400, 500 and 1000 μg/mL) for 24 h. After 24 h, to the untreated cells, 10 μL of cell lysis solution (2% Triton X-100 in PBS) were added; this accounted for the total lactate dehydrogenase (LDH) activity. Cells were centrifuged at 2000× *g* for 5 min at 4 °C, and 100 μL of the supernatant were collected. To this, 900 μL of the LDH estimation (Agappe-11407002, Kerala, India) reaction mixture was added and measured spectrophotometrically at 340 nm for 3 min.

### 2.8. Statistical Analysis

All of the analyses were carried out in triplicate. The values of the *in vitro* antioxidant assays are expressed as the mean ± standard deviation. The other tests were analyzed by one-way ANOVA followed by Tukey’s *post hoc* test. *p* < 0.05 was considered significant.

## 3. Results and Discussion

### 3.1. Metabolite Profile of N. jatamansi

#### 3.1.1. Reversed Phase-HPLC Analysis of Phenolic Compounds

**Figure 1:**
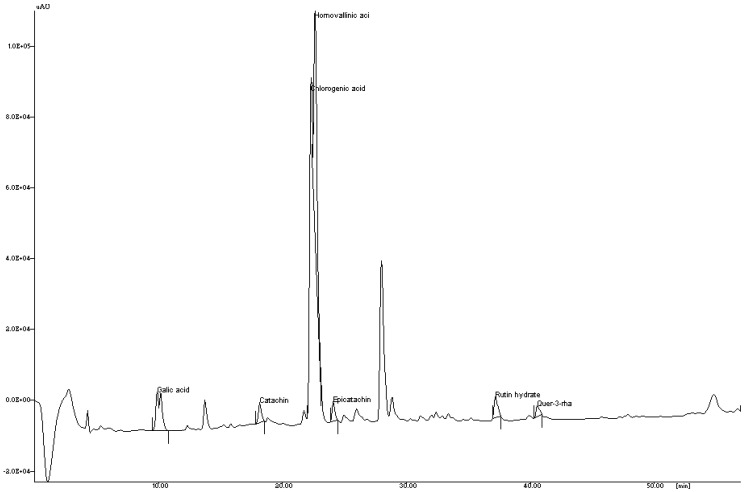
RP-HPLC chromatogram of the identified polyphenols in the ethanolic extract of *N. jatamansi*.

The reversed phase-HPLC analysis aided in the identification (Figure 1) and quantification (Table 1) of polyphenols present in NJE. The identified polyphenols include gallic acid (0.18 mg/g), catechin (4.37 mg/g), chlorogenic acid (19.90 mg/g), homovanillic acid (32.02 mg/g), epicatechin (4.23 mg/g), rutin hydrate (0.08 mg/g) and quercetin-3-rhamnoside (7.13 mg/g). However, a few of the polyphenols could not be identified, due to a lack of standards. The relative concentrations of each identified metabolite was estimated on the basis of the relative standard calibration curve.

**Table 1:** Quantification of the identified polyphenols by RP-HPLC.

No.	Polyphenols	RT (min)	Concentration (mg/g)
1	Gallic acid	9.88	0.18
2	Catechin	18.16	4.37
3	Chlorogenic acid	22.27	19.90
4	Homovanillin	22.60	32.02
5	Epicatechin	24.09	4.23
6	Rutin hydrate	37.17	0.08
7	Quercetin-3-rhamnoside	40.56	7.13

#### 3.1.2. GC-MS Analysis

**Figure 2:**
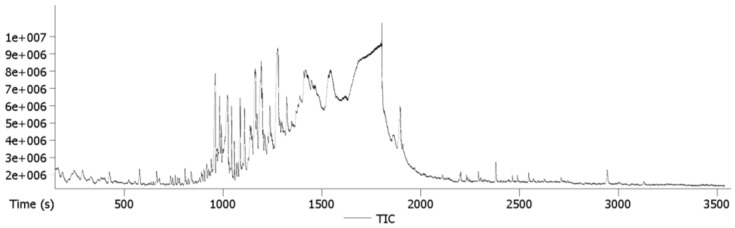
Total ion chromatogram of the hexane fraction of *N. jatamansi*.

The total ion chromatogram (TIC) of the hexane fraction of *N. jatamansi* is shown in Figure 2. The chemical formulae, retention time and mass of the identified compounds are listed in Table 2.

**Table 2:** Phytochemical analysis of the *N. jatamansi* hexane extract.

No.	RT (min)	Compound Name	Chemical Formula	Mass	Area (%)	Hit
1	9.62	Dodecane	C_12_H_26_	170.204	1.615	1
2	11.22	2-Furanmethanol, tetrahydro-5-methyl-*trans*	C_6_H_12_O	116.084	0.031	1
3	13.98	Linalool	C_10_H_18_O	154.135	0.362	1
4	15.30	Benzenemethanol, a-methyl-propanoate	C_11_H_14_O_2_	178.099	0.705	1
5	15.68	1H-Cyclopropa(a)naphthalene, 1a,2,3,5,6,7,7a,7b-octahydro-1,1,7,7a-tetramethyl-(1aR-(1aa,7a,7aa,7ba))	C_15_H_24_	204.188	1.061	1
6	16.02	a-Muurolene	C_15_H_24_	204.188	14.071	1
7	16.39	1H-Cycloprop(e)azulene,1a,2,3,4,4a,5,6,7b-octahydro-1,1,4,7-tetramethyl-(1aR-(1aa,4a,4aa,7ba))	C_15_H_24_	204.188	9.184	5
8	16.69	l-calamenene	C_15_H_22_	202.172	0.065	1
9	17.11	Α-ionone	C_13_H_20_O	192.151	0.643	1
10	17.41	Bicyclo(3.3.1)nonan-2-one,1-methyl-9-(1-methylethylidene)	C_13_H_20_O	192.151	8.329	1
11	17.63	Neoisolongifolene,8,9-dehydro	C_15_H_22_	202.172	2.002	1
12	17.81	(−)-à-Panasinsen	C_15_H_24_	204.188	1.716	1
13	18.13	Neoisolongifolene,8,9-dehydro	C_15_H_22_	202.172	10.86	1
14	18.96	Bicyclo(2.2.2)octa-2,5-diene, 1,2,3,6-tetramethyl	C_12_H_18_	162.141	3.643	1
15	19.15	Isolongifolene,4,5,9,10-dehydro	C_15_H_20_	200.157	1.24	1
16	19.76	*trans*-Nerolidol	C_15_H_26_O	222.198	4.781	1
17	19.88	para-methoxyphenylpiperazine	C_11_H_16_N_2_O	192.1263	5.576	1
18	20.2	Cyclolongifolene oxide, dehydro	C_15_H_22_O	218.167	1.368	1
19	21.51	1(2H)-Naphthalenone,octahydro-4a,8a-dimethyl-7-(1-ethylethyl)-, (4aR-(4aa,7a,8aa))	C_15_H_26_O	222.198	0.329	1
20	22.05	2-tetradecenal	C_14_H_26_O	210.1984	4.873	1
21	30.1	Palmitic acid	C_16_H_32_O_2_	256.24	1.537	1
22	31.64	Oleic acid	C_18_H_34_O_2_	282.256	5.499	1
23	32.86	2,8,9-Trioxa-5-aza-1-silabicyclo(3.3.3)undecane,1-methoxy	C_7_H_15_NO_4_Si	205.077	0.165	1
24	37.22	Tridecanoic acid, methyl ester	C_14_H_28_O_2_	228.209	0.625	1
25	39.71	Heptacosane	C_27_H_56_	380.438	2.664	1

### 3.2. In Vitro Antioxidant and Free Radical Scavenging Activities

The DPPH free radical scavenging assay results represent a strong effect of the extract to scavenge free radicals. The percent scavenging activity of the extracts showed an increase with the subsequent increase in the concentration of the extract. The 70% ethanol extract was more potent than the hexane fraction, and the IC_50_ values for DPPH, ABTS^+^, superoxide radical scavenging, metal chelation and ferric reducing antioxidant activities were found to be 222.22 ± 7.4 μg/mL, 13.90 ± 0.5 μg/mL, 113.81 ± 4.2 μg/mL, 948 ± 21.1 μg/mL and 12.3 ± 0.43 mg FeSO_4_E/g of extract, respectively. However, the hexane fraction also exhibited antioxidant activity with IC_50_ values of 432.68 ± 13.7 μg/mL for DPPH, 23.57 ± 1.4 μg/mL for ABTS^+^, 255.72 ± 9.7 μg/mL for superoxide radical scavenging, 1211 ± 27.8 μg/mL for metal chelation and 45.62 ± 1.34 mg FeSO_4_E/g of extract for the ferric-reducing antioxidant assay (Table 3).

**Table 3:** Polyphenol and flavonoid content, antioxidant and free radical scavenging activities of ethanolic extract of *Nardostachys jatamansi* (NJE) and the hexane fraction (NJH). Values are represented as the means ± SD of the triplicate determination. ABTS, 2,2′-azino-bis(3-ethyl benzothiazoline-6-sulfonic acid) diammonium salt. CE, catechin equivalents.

Assay	Ethanolic Extract (NJE)	Hexane Extract (NJH)
Total polyphenolic content	53.06 ± 2.2 mg GAE/g of extract	13.87 ± 1.3 mg GAE/g of extract
Total flavonoids	25.303 ± 0.9 mg CE/g of extract	4.58 ± 0.3 mg GAE/g of extract
DPPH radical scavenging assay (IC_50_)	222.22 ± 7.4 μg/mL	432.68 ± 13.7 μg/mL
Metal chelation (IC_50_)	948 ± 21.1 μg/mL	1211 ± 27.8 μg/mL
ABTS (IC_50_)	13.90 ± 0.5 μg/mL	23.57 ± 1.4 μg/mL
Superoxide (IC_50_)	113.81 ± 4.2 μg/mL	255.72 ± 9.7 μg/mL
Anti-lipid peroxidation (IC_50_)	465.11 ± 14.3 μg/mL (brain)	587.53 ± 17.6 μg/mL (brain)
	539.08 ± 18.9 μg/mL (liver)	685.15 ± 13.4 μg/mL (liver)
Ferric-reducing antioxidant power	12.3 ± 0.43 mg FeSO_4_E/g of extract	45.62 ± 1.34 mg FeSO_4_E/g of extract

The 70% ethanol extract exhibited strong antioxidant capacity in comparison to that of the hexane extract. Hence, the 70% ethanol extract was further used to analyze the protective effect against AAPH-induced oxidative damage of biomolecules.

#### Polyphenol and Flavonoid Contents

NJE showed a high content of polyphenols, 53.06 ± 2.2 mg GAE/g of the extract (gallic acid equivalent), and flavonoids, 25.303 ± 0.9 mg CE/g of the extract (catechin equivalents), whereas NJH showed 13.87 ± 1.3 mg GAE/g of the extract of polyphenols and 4.58 ± 0.3 mg GAE/g of the extract of flavonoids (Table 3).

### 3.3. Protective Properties against Biomolecules Oxidation

#### 3.3.1. NJE Inhibits AAPH-Induced DNA Damage

**Figure 3:**
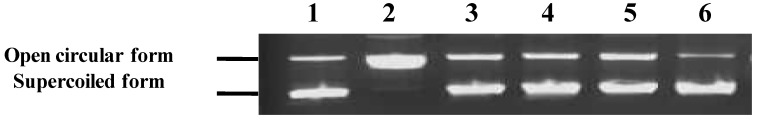
Inhibition of DNA damage by NJE. Lane 1, plasmid DNA (pBR322); Lane 2, DNA + 2,2′-azobis(2-methylpropionamidine) dihydrochloride (AAPH); Lane 3, DNA + AAPH + 5 μg extract; Lane 4, DNA + AAPH + 10 μg extract; Lane 5, DNA + AAPH + 15 μg extract; Lane 6, DNA + AAPH + 10 μg GA.

AAPH is a water-soluble initiator, which decomposes at physiological temperature, producing alkyl radicals, which react with oxygen to form alkyl peroxyl radicals to initiate protein oxidation [37]. The protective potential of the plant extract against DNA damage induced by AAPH (200 mM) was measured using pBR322 DNA. As shown in Figure 3, the plasmid DNA was mainly of the supercoiled form (bottom band) and open circular form (top band) in the absence of AAPH (Lane 1). With the addition of 10 mM AAPH, the supercoiled form decreased and converted into the open circular form (Lane 2). The addition of the extract significantly inhibited the formation of the open circular form as compared to the positive control (85%; 15 μg/mL extract). Gallic acid at 5 μg/mL also showed significant protection against DNA damage (95%).

#### 3.3.2. NJE Inhibits Protein Oxidation

**Figure 4:**
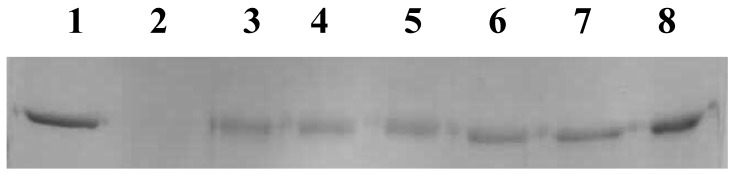
Inhibition of protein oxidation by NJE. Lane 1, BSA; Lane 2, BSA + AAPH; Lane 3, BSA + AAPH + 5 μg extract; Lane 4, BSA + AAPH + 10 μg extract; Lane 5, BSA + AAPH + 15 μg extract; Lane 6, BSA + AAPH + 20 μg extract; Lane 7, BSA + AAPH + 25 μg extract; Lane 8, BSA + AAPH + 10 μg GA.

The protective property of the plant extract against protein oxidation was determined by the oxidation of BSA initiated by AAPH. After incubation for 2 h at 37 °C, the samples were analyzed using SDS-PAGE. The results demonstrated that the plant extract showed 50% protection against BSA oxidation (Lanes 3, 4, 5, 6, 7) in a dose-dependent manner. In Lane 2 (positive control), in the presence of the radical inducer AAPH (200 mM), BSA was completely degraded, whereas gallic acid at 10 μg/mL showed a complete protective effect (Figure 4).

#### 3.3.3. Inhibition of Lipid Peroxidation

Further, the potential of 70% ethanol extract to inhibit lipid peroxidation induced by AAPH (a peroxyl radical inducer) in rat liver and brain homogenate was analyzed by the TBARS assay. The IC_50_ values of NJE for inhibiting lipid peroxides in the brain and liver are 465.11 ± 14.3 μg/mL, 539.08 ± 18.9 μg/mL, respectively, and for NJH are 587.53 ± 17.6 μg/mL (brain) and 685.15 ± 13.4 μg/mL (liver) (Table 3).

#### 3.3.4. Inhibition of Reactive Oxygen Species

**Figure 5:**
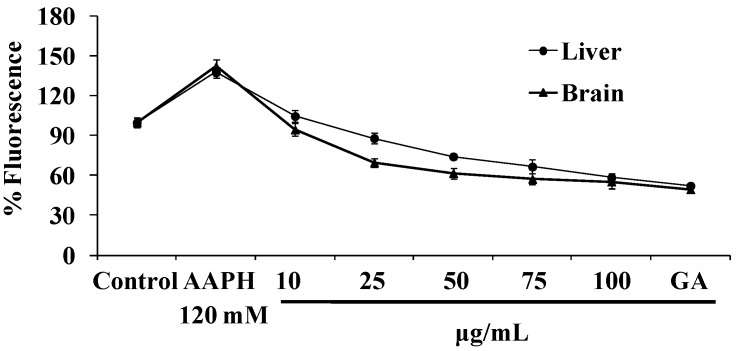
Antioxidant effects of NJE and gallic acid on AAPH-induced ROS generation.

This assay was performed to check the protective efficacy of the plant extract against intracellular ROS generation. AAPH caused a significant increase in ROS production in comparison with that of the control. NJE at 100 μg/mL was effective at combating ROS and showed 58% and 55% ROS inhibition in the liver and brain homogenates (Figure 5).

#### 3.3.5. NJE Inhibits Protein Carbonyl Content

**Figure 6:**
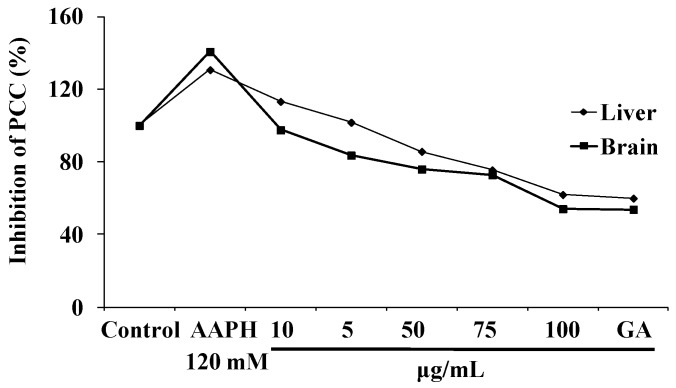
Inhibitory effects of NJE and gallic acid on AAPH-induced protein carbonyl formation.

The protein carbonyl formation increases drastically under various pathological conditions of oxidative stress [38]. AAPH significantly increased the formation of protein carbonyls in comparison to that of the control. The increasing concentration of the extract was effective at inhibiting the protein carbonyls formed with 100 μg/mL of NJE, showing a 61% and 54% inhibitory effect on protein carbonyls formed in the liver and brain homogenates (Figure 6).

### 3.4. Cytotoxicity of NJE by the Plasma Membrane Leakage Assay

**Figure 7:**
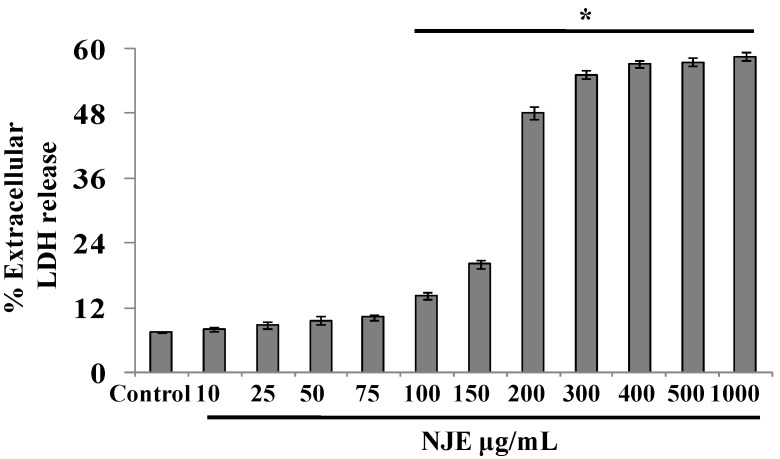
Effects of NJE on LDH release in SH-SY5Y neuronal cells (* *p* < 0.05 *vs.* control).

Cell damage was evaluated by measuring the leakage of intracellular LDH into the medium. The extract was not toxic up to 75 μg/mL. However, pretreatment of the cells above 100 μg/mL exhibited cell damage, and an increased LDH release was observed (Figure 7).

## 4. Discussion

Modern lifestyle, including the fast pace of life and reduced physical activity, seems to have a direct, as well as indirect effect on human health, especially mental and physical stress. The first and foremost manifestation is anxiety. Excessive anxiety can prove to be disabling and could lead to other neuropsychiatric disorders. There are several reports demonstrating that physical/mental stress leads to oxidative stress, which, in turn, becomes a cause for more serious illness [39,40].

In the present investigation, preliminarily, we analyzed the phytochemicals present in *N. jatamansi*. Reversed phase-HPLC analysis aided in quantifying the polyphenols present in NJE. The identified polyphenols contribute to the enhanced antioxidant potency of the herb and help in scavenging ROS. In plants, polyphenols and flavonoids serve as protectors against a wide variety of environmental stresses, while, in humans, flavonoids appear to function as “biological response modifiers”. The broad therapeutic effects of polyphenols and flavonoids can be largely attributed to their antioxidant properties [7]. Polyphenols and flavonoids are naturally-occurring antioxidant substances, which exhibit myriad pharmacological actions on the central nervous system and are effective at managing anxiety and depression [41]. Reports demonstrated that polyphenols, such as gallic acid, catechin, chlorogenic acid and quercetin-3-rhamnoside, exhibit anxiolytic effects [42]. GC-MS analysis aided in the identification of an array phytochemicals, such as fatty acids, sesquiterpenes, alkane hydrocarbons and esters. Among the identified compounds, alpha-muurolene, a sesquiterpene, was found to be abundant. Sesquiterpenes have been ascribed as potent antioxidants [43]. Another identified metabolite, linalool, a monoterpene, has been used as a sleep aid and is effective in the treatment of psychosis and anxiety [44]. Omega 3-fatty acids and long-chain polyunsaturated fatty acids have been implicated with diminishing inflammation [45] and reduce the risk of thrombosis [46].

Polyphenols and flavonoids are gaining importance for their antioxidant properties, as they are effective hydrogen donors [47]. These natural antioxidants provide health benefits associated with preventing damage due to biological degeneration and any oxidative onslaught [34]. Previous studies also reported that the dried roots of *N. jatamansi* contain sesquiterpenes, like jatamansone, spirojatamol, patchouli alcohol, norseychelanone, jatamol A and B, lignans and neolignans, like virolin, pinoresinol, jatamansic acid, and terpenic coumarins, like oroselol and jatamansin. The essential oil is composed of sesquiterpenoids and coumarins. Jatamansone is the principle sesquiterpenoid and is attributed with rendering majority of the biological activity [48,49].

The free radical scavenging activity of *N. jatamansi* was evaluated by an array of antioxidant assays. The DPPH free radical scavenging assay is extensively employed to determine the antioxidant activities of different samples in a short time. This suggests that the plant extract contains compounds that are capable of donating hydrogen to a free radical in order to remove the odd electron, which is responsible for the radical’s reactivity [50]. The ABTS***^+^*** radical scavenging assay reflects the capacity of an antioxidant species to donate electrons or hydrogen atoms to inactivate this radical cation. The reducing power of a compound serves as a significant indicator of its potential antioxidant activity [51]. The ferric-reducing antioxidant power assay is described as a rapid and novel method for assessing the antioxidant power of various samples. Fe^3+^ reduction is often used as an indicator of electron donating activity, which is an important mechanism of phenolic antioxidant action and can be strongly correlated with other antioxidant properties [52]. The superoxide anion radical is one of the strongest reactive oxygen species generated among the free radicals. Thus, the antioxidant activity is attributed to various mechanisms, like the prevention of chain initiation, binding of transition metal ion catalysts, prevention of continued hydrogen abstraction, reductive capacity, radical scavenging activity and decomposition of peroxides [53,54]. In the present study, the results suggest that 70% ethanol extract is a potent scavenger of free radicals compared to the hexane extract. This might be due to the diverse content of phytochemicals in the 70% ethanol fraction of *N. jatamansi*.

The excessive production of free radicals, collectively called ROS, is known to cause oxidation of biomolecules and is observed in degenerative diseases and ageing. Thus, the inhibition of oxidation of these biomolecules may be vital for the alleviation of an array of diseases. Damage to DNA is known to alter replication and transcription, causing cell death or mutations. Oxidative modifications of DNA have been suggested to contribute to aging and various diseases, including cancer and chronic inflammation [55]. Lipid peroxidation is a free radical chain reaction, which arises from the oxidative conversion of polyunsaturated fatty acids by HO^•^ to lipid peroxides, which, in turn, can damage biological membranes. The malondialdehyde level is widely utilized as a marker of lipid peroxidation in states of elevated oxidative stress. Thus, it can be correlated to the identified metabolites serving as antioxidants, being mainly capable of scavenging these free radicals and thereby preventing DNA damage, protein oxidation and lipid peroxidation. The results are suggestive of the fact that the plant extract is effective at the prevention of lipid peroxidation, a common causative agent in cell membrane disruption and cell damage [56].

The protective effect of antioxidants on cells is thought to reside in their ability to reduce the level of intracellular ROS generation [57]. The fluorescent probe 2′,7′-dichlorodihydro fluorescein diacetate (DCFH-DA) is used to measure ROS that crosses cell membranes and that are enzymatically hydrolysed to nonfluorescent DCFH. In the presence of ROS, DCFH is oxidized to a highly fluorescent compound, 2′,7′-dichlorofluorescein (DCF), and measured to quantify the ROS generation [58]. AAPH, a peroxyl radical generator, caused an increase in ROS production that was alleviated by NJE dose dependently.

The oxidative damage to the proteins caused by free radicals has been shown to play a significant role in aging and several pathological events [59]. The major molecular mechanism leading to structural modifications in proteins is free radical-mediated protein oxidation characterized by carbonyl formation. The measurement of protein carbonyls is used to assess the oxidative damage of proteins [60]. As observed, NJE inhibited protein carbonyl (PCO) formation, confirming its antioxidative role and demonstrating that it could be a good source of antioxidants and could protect against oxidative insults. The cytotoxic effect of the extract assessed by the LDH assay on SH-SY5Y cells showed that the extract is non-toxic up to the dose of 75 g/mL and could be used to further verify the antioxidant effects at the cellular level.

## 5. Conclusions

Oxidative stress causes cellular damage and subsequent cell death. The results obtained suggests that *Nardostachys jatamansi* rhizome contains good amounts of polyphenols, flavonoids and sesquiterpenes and thereby exhibits high antioxidant and free radical scavenging activities. The extract was found to chelate iron, scavenge superoxide radical and has a high reducing power. It inhibited protein oxidation, DNA damage and decreased the malondialdehyde level. The extract effectively scavenged ROS and inhibited protein carbonyls. Hence, these *in vitro* assays support the idea that *Nardostachys jatamansi* can be a good source of natural antioxidants to alleviate oxidative stress-mediated disorders.
